# Aortic isthmus Doppler hemodynamics and ımpacts on perinatal outcomes in pregestational and gestational diabetes mellitus

**DOI:** 10.1007/s00404-026-08378-3

**Published:** 2026-03-19

**Authors:** Zeynep Seyhanli, Gulsan Karabay, Ahmet Arif Filiz, Dilara Duygulu Bulan, Recep Taha Agaoglu, Gizem Aktemur, Betul Tokgoz Cakir, Kadriye Yakut Yucel, Zehra Vural Yilmaz

**Affiliations:** Department of Obstetrics and Gynecology, Division of Perinatology, Ankara Etlik City Hospital, Ankara, Turkey

**Keywords:** Aortic isthmus, Doppler, Gestational diabetes mellitus, Composite adverse perinatal outcomes

## Abstract

**Background/Aim:**

To assess the sensitivity of the fetal aortic isthmus (AoI) Doppler changes in predicting the adverse perinatal outcome with pregestational and gestational diabetes mellitus compared with other Doppler parameters.

**Materials and methods:**

This prospective case–control study was undertaken between Agust 2023 and May 2024 in Ankara Etlik City Hospital perinatology department. Maternal age, gravidity, parity, maternal body mass index (BMI), gestational age at ultrasonographic examination, fetal AoI, umbilical artery (UA), middle cerebral artery (MCA), and uterine artery (UtA) Doppler parameters were compared between four groups: Pregestational diabetes mellitus (PGDM) (*n* = 30), diet-regulated gestational diabetes mellitus (DRGDM) (*n* = 30), insulin-regulated GDM (IRGDM) (*n* = 30), and controls (*n* = 75). HbA1c values were evaluated only in pregnancies complicated by diabetes mellitus. The composite adverse perinatal outcomes (CAPO) was defined as Apgar score at 5 min < 7, cord blood pH < 7, sepsis, phototherapy for neonates, respiratory distress syndrome (RDS), mechanical ventilation, neonatal intensive care unit (NICU) admission, hypoglycemia.

**Results:**

Maternal age and BMI levels were significantly higher in the PGDM group (*p* < 0.001). HbA1c levels were significantly higher in the PGDM group compared to other diabetic groups (*p* < 0.001). While most Doppler parameters were similar across groups, PGDM cases had higher rates of prematurity (40%), cesarean delivery (90%), NICU admission (60%), and CAPO (63.3%) compared to controls (*p* < 0.01 for all). HbA1c was positively correlated with MCA S/D, AoI-PSV, and AoI S/D (*p* < 0.05). ROC analysis showed that AoI Ta-max and EDV had modest predictive value for CAPO (AUC = 0.627 and 0.638, respectively; *p* < 0.05).

**Conclusions:**

The AoI Doppler flow measurements may serve as a potential adjunctive marker in the assessment of adverse perinatal outcomes in pregnancies complicated by diabetes mellitus. However, further large-scale prospective studies are warranted to validate its clinical applicability and establish its role in routine obstetric practice.

## What does this study adds to the clinical work

**Table:** 

Aol Doppler assessment may provide complementary information when integrated with established clinical and sonographic parameters, improving risk stratification and follow-up in high-risk diabetic pregnacies.	

## Introduction

Pre-gestational diabetes (PGDM) is a complex disease that poses both maternal and fetal risks and is increasingly common. PGDM is diabetes that exists before pregnancy, and the most common types are Type 1 (T1DM) and Type 2 (T2DM) diabetes mellitus which are associated with an increased risk of complications during pregnancy [1, 2]. Between 0.5 and 2.4% of all pregnancies globally are affected by both T1DM and T2DM type 2 [3].

Gestational diabetes mellitus (GDM), defined as glucose intolerance first recognized during pregnancy, is likewise associated with adverse maternal and neonatal outcomes, including hypertensive disorders, cesarean delivery, shoulder dystocia, prematurity, and neonatal respiratory morbidity [4, 5]. Although PGDM and GDM differ in pathophysiology and severity, both conditions share overlapping adverse perinatal outcomes, underscoring the importance of refined fetal surveillance strategies in diabetic pregnancies [5, 6].

Hyperglycemia throughout pregnancy heightens the risk of maternal and fetal/neonatal complications. Maintaining glycemic control is crucial in order to prevent a variety of problems, and it is the fundamental aspect of treatment. Consequently, it is crucial to improve blood glucose management in both PGDM and GDM. HbA1c levels are a way to assess blood glucose regulation in women with PGDM. The recommended thresholds are less than 6.5% during the first trimester and less than 6.0% throughout the second and third trimesters [4].

Neurocognitive disorders, which are thought to be related to the degree of fetal exposure to hyperglycemia and the effect of hyperglycemia on the developing central nervous system, can be seen in T1DM and, similarly, in GDM treated with insulin, which is associated with neonatal hypoglycemia and worse neonatal neural adaptation [7, 8].

The aortic isthmus (AoI) is the segment between the point where the left subclavian artery origins and the point where the ductus arteriosus connects to the aorta, also it is the sole arterial connection between the two fetal circulatory systems that are positioned in parallel. Studies have shown that AoI Doppler flow information can be an indicator of fetoplacental hemodynamic disturbances and provide an additional contribution to fetal cardio-circulation [9–12]. Subsequently, a thorough assessment of AoI blood flow was conducted, and many parameters associated with it were measured to establish normal values [13, 14]. AoI Doppler has been suggested for its potential to indicate the presence of fetal hemodynamic compromise and to predict perinatal outcomes and long-term neurodevelopment [9, 10, 15]. Nevertheless, to the best of our knowledge, there is a scarcity of studies investigating the correlation between the aortic isthmus and perinatal outcomes in pregnant women with DM [16–18].

The objective of this prospective study was to assess the correlation between AoI Doppler flow measurements and perinatal outcomes in pregnancies complicated by PGDM and GDM, thereby filling an essential gap in the current literature.

## Materials and methods

The prospective case–control study was conducted at the maternal fetal medicine clinic of Ankara Etlik City Hospital between August 2023 and May 2024. Gestational age-matched PGDM, GDM and control pregnant women who met the study criteria were included in the study. Nevertheless, the study excluded pregnant women with the following conditions: preeclampsia, preterm premature rupture of membranes, fetal congenital and chromosomal anomalies, smoking, multiple pregnancies, administration of tocolytic and antenatal corticosteroid treatment, chronic maternal diseases other than diabetes mellitus. This study was approved by the ethics committee of Ankara Etlik City Hospital with the approval number: AEŞH-EK1-2023–358.

Maternal age, gravidity, parity, BMI, gestational age at the time of ultrasound examination, AoI peak systolic velocity (PSV), end-diastolic velocity (EDV), the ratio of peak systolic to end-diastolic velocity (S/D), pulsatility index (PI), resistance index (RI) and time-averaged maximum velocity (Ta-max) velocities, umbilical artery pulsatility index (UA PI), middle cerebral artery pulsatility index (MCA PI), uterine artery pulsatility index (UtA PI), cerebroplacental ratio (CPR) which is calculated as the MCA PI / UA PI, cerebralplacental uterin ratio (CPUR) which is calculated as the ratio of CPR to UtA PI and the single deepest pocket (SDP) were compared between the three groups. Following that, the group diagnosed with GDM was subdivided into two subgroups: DRGDM, IRGDM. The follow-up, treatment plan and birth of pregnant women diagnosed with PGDM and GDM were carried out by a multidisciplinary team. HbA1c measurement was routinely performed in pregnancies complicated by pregestational or gestational diabetes mellitus as part of standard clinical follow-up. HbA1c was not measured in the control group, as it is not routinely assessed in normoglycemic pregnancies in our clinical practice.

The diagnosis of GDM was made if any of the fasting plasma blood glucose values were ≥ 92 mg/dL, the first hour value was ≥ 180 mg/dL, and the second hour value was ≥ 153, respectively, after the 75-g OGTT [4].

**Fig. 1:**
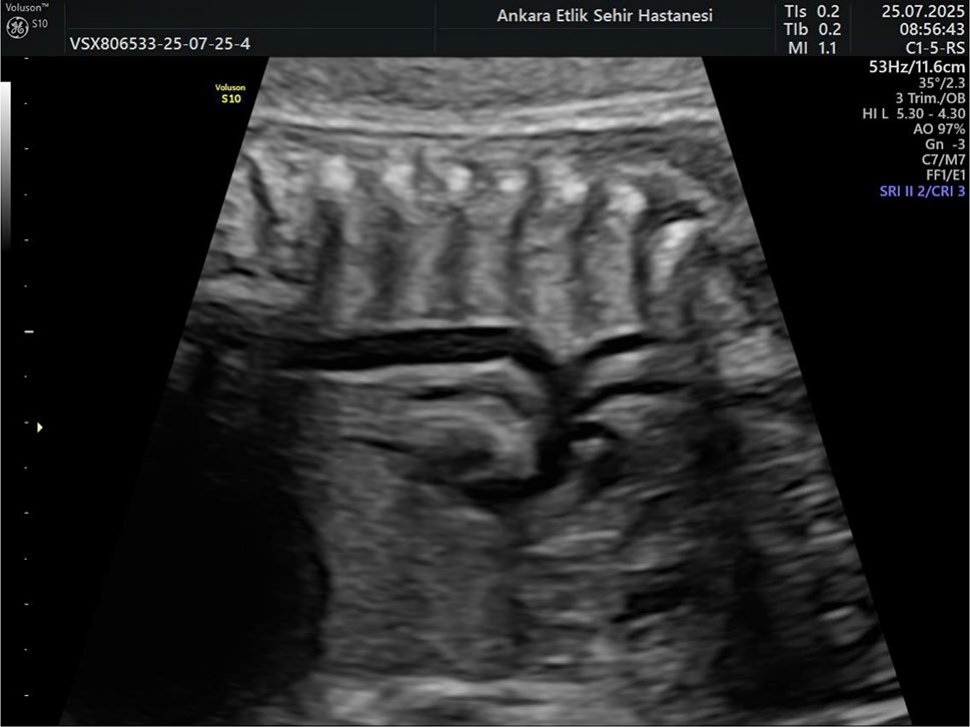
Aortic isthmus in the sagittal plane obtained during third trimester fetal ultrasound

**Fig. 2:**
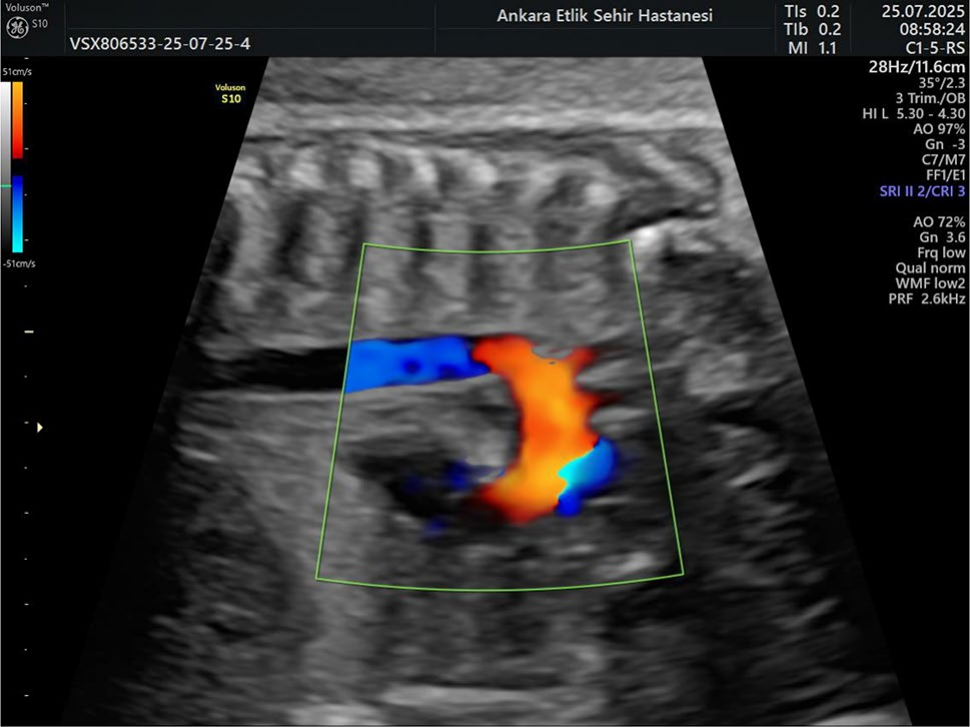
Sagittal color Doppler ultrasound image of the fetal aortic isthmus showing blood flow between the aortic arch and descending aorta

**Fig. 3:**
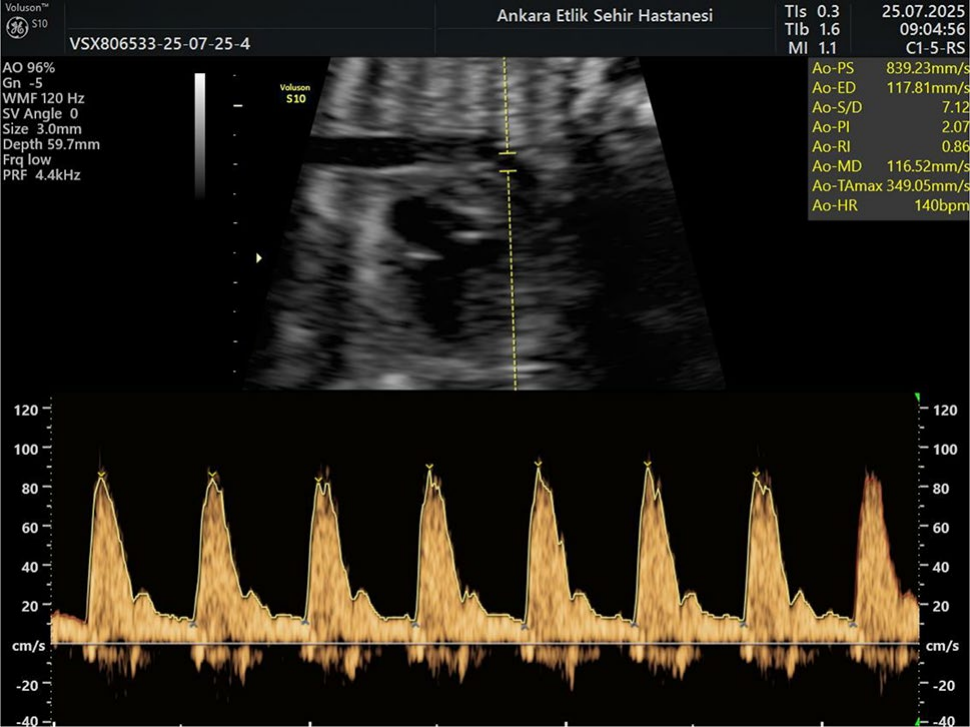
Pulsed-wave Doppler assessment of the fetal aortic isthmus showing forward flow during systole and early diastole in a sagittal view

Ultrasonographic examination was performed utilizing a Voluson S10 machine equipped with a 2–5 MHz convex probe during the period of 28–37 weeks of gestation. All ultrasound and Doppler examinations were performed by a single experienced maternal–fetal medicine specialist using a standardized imaging protocol. This week interval was selected for the measurements because it follows the week interval for GDM screening and fetal complications associated with GDM typically manifest after the second trimester. The aortic isthmus was visualized in the sagittal plane using 2D ultrasound during the third trimester, as illustrated in Fig. 1. Doppler evaluation of the AoI, a few millimeters beyond the origin of the left subclavian artery and just above the junction of the ductus arteriosus, was obtained after visualizing the aortic arch in the sagittal view [19] (Fig. 2). The aortic isthmus waveform can be derived from the three vessels and the trachea view of the aortic-ductus arteriosus connection [20, 21]. The color Doppler maximal velocity setting was modified to high velocity, but the high-pass filter was configured at 50 Hz and the energy output levels were kept below 50 mW/cm2.The scanning plane was calibrated to achieve an insonation angle as close to 0 degrees as possible, and always less than 30 degrees. Recordings were conducted while fetal movements were not present (Fig. 3).

The Aortic isthmus flow index (IFI) was computed using the methodology established by Fouron, applying pulsed-wave Doppler flow analysis asthe sum of systolic and diastolic velocity–time integrals to the systolic velocity–time integral: IFI = S + D/S [22].

Composite adverse perinatal outcomes (CAPOs) include the presence of at least one of the following adverse outcomes: 5th-minute APGAR score < 7, respiratory distress syndrome (RDS), cord blood pH < 7, need for mechanical ventilation, neonatal hypoglycemia, need for phototherapy, neonatal intensive care unit (NICU) admission, and neonatal sepsis.

### Statistical analysis

IBM Corporation SPSS version 22.0 (IBM Corporation, Armonk, NY, USA) was used to conduct the statistical analysis. The conformance to the normal distribution was examined using the Kolmogorov–Smirnov test. For continuous variables with a normal distribution, descriptive statistics are displayed as "mean ± standard deviation"; for those without, they are displayed as "median (min–max value)." When comparing more than two groups, the Analysis of Variance (Anova) test was employed. The number of groups was taken into consideration while determining the Anova test's statistical significance. Fisher's exact test or the chi-squared test were used to compare categorical variables. The independent sample t-test and the Mann–Whitney U test were used to examine continuous variables that were and were not regularly distributed. Univariate and multivariate analyses were performed to investigate potential co-founders. For all tests, a P-value of less than 0.05 was considered statistically significant.

An a priori power analysis was conducted using the G*Power software (version 3.1.9.7; Heinrich Heine University Düsseldorf, Düsseldorf, Germany) to determine the minimum required sample size based on data from relevant literature [16]. The effect size derived from this reference study was large (Cohen’s d = 1.59). Assuming a two-sided significance level of α = 0.05, the analysis indicated that a minimum of 12 participants per group would be sufficient to achieve adequate statistical power.

## Results

The study was conducted with four groups of 30 (18.2%) pregnant women each with PGDM, DRGDM, IRGDM and 75 (45.5%) pregnant control women. A total of 165 patients were included in the study. Maternal, perinatal, Doppler ultrasound, and neonatal parameters were compared between these groups.

Perinatal characteristics such as doppler flow measurements of the AoI, UA and UtA as well as SDP and maternal characteristics of the pregnant women classified into 4 groups are shown in Table 1. Maternal age and BMI were significantly higher in the PGDM group compared to the control group (*p* < 0.001). HbA1c levels were significantly different among the diabetic groups (*p* < 0.001), with the highest values observed in the PGDM group. There were no significant differences among groups in terms of gestational age at ultrasound, gravidity, or parity. Doppler parameters including AoI PI, RI, PSV, EDV, S/D ratio, and Ta-max did not show significant differences among the groups. Similarly, the MCA-PI, UA-PI, UtA-PI, CPR, and CPUR values were comparable (*p* > 0.05). However, SDP of amniotic fluid was significantly lower in the control group compared to diabetic groups (*p* < 0.001) (Table 1).

**Table 1:** Comparison of maternal—perinatal characteristics of the pregnant women and aortic isthmus, umbilical, uterine doppler flow measurements and amniotic fluid volume of the fetuses

	PGDM *n* = 30	DRGDM *n* = 30	IRGDM *n* = 30	Control *n* = 75	*p value*
*Maternal age (year)*	33.4 ± 4.9	29.7 ± 4.9	31.4 ± 5	28.1 ± 4.9	** < 0.001**^**a**^
*Gravida*	3 (1–6)	2.5 (1–5)	2 (1–7)	2 (1–6)	0.124^b^
*Parity*	1 (0–3)	1 (0–3)	1 (0–3)	1 (0–4)	0.362^b^
*BMI*	33.4 ± 4	31.8 ± 6.3	32.9 ± 4.7	29.5 ± 4.5	** < 0.001**^**a**^
*Gestational age at USG examination (week)*	32 (28 – 37)	35 (28 -37)	33 (28 – 37)	33 (28 – 37)	0.057^b^
*HbA1c*	6.09 ± 1.38	5.02 ± 0.34	5.65 ± 0.81	-	** < 0.001**^**a**^
*AoI-PI*	2.39 ± 0.4	2.28 ± 0.33	2.33 ± 0.4	2.3 ± 0.3	0.748^a^
*AoI-RI*	0.87 ± 0.02	0.86 ± 0.02	0.85 ± 0.06	0.84 ± 0.14	0.731^a^
*AoI-PSV*	94.4 ± 37	85.7 ± 33.3	92.2 ± 34	86.8 ± 41.3	0.727^a^
*AoI-EDV*	11.3 ± 3.2	11.5 ± 4.7	12.6 ± 6.33	12.8 ± 11.4	0.805^a^
*AoI S/D*	8.23 ± 1.89	7.67 ± 1.71	7.82 ± 2.29	7.82 ± 2.21	0.746^a^
*AoI-Ta max*	34.5 ± 12.5	32.1 ± 14.6	34.6 ± 12.7	35.1 ± 24.3	0.915^a^
*IFI*	1.12 ± 0.02	1.13 ± 0.02	1.14 ± 0.06	1.15 ± 0.14	0.731^a^
*MCA-PI*	1.72 ± 0.31	1.5 ± 0.39	1.58 ± 0.32	1.65 ± 0.36	0.08^a^
*UA-PI*	0.89 ± 0.15	0.84 ± 0.16	0.82 ± 0.18	0.89 ± 0.18	0.175^a^
*UtA-PI*	0.8 ± 0.28	0.85 ± 0.44	0.86 ± 0.29	0.81 ± 0.29	0.822^a^
*CPR*	2.38 ± 0.93	2.13 ± 0.84	2.18 ± 1.07	2.26 ± 1.05	0.763^a^
*CPUR*	3.64 ± 0.93	3.33 ± 2.21	3.47 ± 3.35	3.55 ± 3.15	0.978^a^
*SDP*	65 ± 22	66 ± 22	63 ± 16	52 ± 10	** < 0.001**^**a**^

Data are expressed as mean ± *SD or median (min–max) where appropriate. *

*A p value of* < *0.05 indicates a significant difference and statistically significant p-values are in bold. Comparisons involving HbA1c were performed only among diabetic groups*

^a^Analysis of variance with Bonferroni test,

^b^Analysis of variance with Tamhane’s test, *PGDM* pregestational diabetes mellitus, *DRGDM* diet regulated gestational diabetes mellitus, *IRGDM* insulin regulated gestational diabetes mellitus, *BMI* body mass index, *HbA1c* hemoglobin A1c, *AoI* aortic isthmus, *PI* pulsatility index, *RI* resistive index, *PSV* peak systolic velocity, *EDV* end diastolic velocity, *S/D* systolic/diastolic ratio, *Ta max* time-averaged maximum velocity *IFI* aortic isthmus flow index, *MCA* middle cerebral artery, *UA*: umbilical artery, *UtA* uterine artery, *CPR* cerebroplacental ratio, *CPUR* cerebral-placental-uterine ratio, *SDP* single deepest pocket for amniotic fluid

The neonatal outcomes and birth characteristics of the newborns were analyzed separately by group and are shown in Table 2. The median gestational age at delivery was significantly lower in the diabetic groups compared to the control group (*p* < 0.001). Prematurity was more common in the PGDM group (40%) than in controls (10.7%, *p* = 0.007). Cesarean delivery rate was highest in the PGDM group (90%, *p* = 0.004). NICU admission was significantly more frequent in PGDM cases (60%) compared to the control group (10.7%, *p* < 0.001). RDS in newborns and the need for mechanical ventilation are more common in the diabetic group (*p* = 0.003, 0.013 respectively). However, no significant difference was found between the diabetes subgroups. CAPO were significantly more common in the PGDM group (63.3%) compared to controls (10.7%, *p* < 0.001). Neonatal hypoglycemia was reported only in PGDM and IRGDM groups (each 6.7%). A statistically significant difference in neonatal hypoglycemia was observed between the groups (*p* = 0.046). Neonatal sepsis was not observed in any of the newborns in the groups (Table 2).

**Table 2:** Birth characteristics and neonatal outcomes of the newborns

	PGDM *n* = 30	DRGDM *n* = 30	IRGDM *n* = 30	Control *n* = 75	*p-value*
Gestational age at delivery (week)	37 (32–40)	37 (34–40)	37 (34–39)	39 (35–41)	** < 0.001**^**a**^
Prematurity (< 37 weeks)	12 (40%)	8 (26.7%)	8 (26.7%)	8 (10.7%)	**0.007**^**b**^
Cesarean section	27 (90%)	18 (60%)	23 (76.7%)	42 (56%)	**0.004**^**b**^
Birth weight (gram)	3309 ± 588	3212 ± 575	3373 ± 473	3161 ± 432	0.203^c^
Phototherapy for neonates	6 (20%)	0 (0%)	0 (0%)	2 (2.7%)	0.294^b^
Neonatal sepsis	0 (0%)	0 (0%)	0 (0%)	0 (0%)	NA
Apgar score at 1st minute	8.5 (6–9)	9 (6–9)	9 (7–9)	9 (5–9)	0.512^a^
Apgar score at 5th minute	9 (7–10)	10 (8–10)	10 (8–10)	10 (8–10)	0.083^a^
NICU admission	18 (60%)	8 (26.7%)	8 (26.7%)	8 (10.7%)	** < 0.001**^**b**^
Neonatal hypoglycemia	2 (6.7%)	0 (0%)	2 (6.7%)	0 (0%)	**0.046**^**b**^
Umbilical cord pH	7.33 ± 0.07	7.33 ± 0.1	7.35 ± 0.09	7.37 ± 0.1	0.662^c^
CAPO	19 (63.3%)	9 (30%)	8 (26.7%)	8 (10.7%)	** < 0.001**^**b**^

Data are expressed as mean ± *SD, median (min–max) or number (percentage) where appropriate. *

A p value of < *0.05 indicates a significant difference and statistically significant p-values are in bold*

^a^Analysis of variance with Tamhane’s test,

^b^Pearson chi-square,

^c^Analysis of variance with Bonferroni test

*PGDM* pregestational diabetes mellitus, *DRGDM* diet regulated gestational diabetes mellitus, *IRGDM* insulin regulated gestational diabetes mellitus, *NICU* neonatal intensive care unit, *CAPO* Composite adverse perinatal outcome, *NA* not applicable

PGDM vs DRGDM (p = 0.010), PGDM vs IRGDM (p = 0.004), PGDM vs control (p < 0.001), DRGDM vs control (p = 0.015), IRGDM vs control (p = 0.013)

**Fig. 4:**
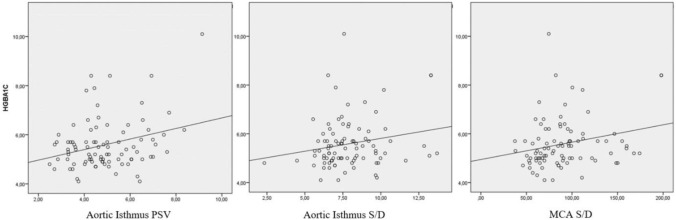
Scatterplot of aortic isthmus PSV, S/D and middle cerebral artery S/D according to HgA1c

The correlation between HbA1c and fetal Doppler parameters was investigated and is shown in Table 3. HbA1c was positively correlated with MCA-S/D (*r* = 0.289, *p* = 0.006), AoI-PSV (*r* = 0.238, *p *= 0.024), and AoI S/D (*r* = 0.200, *p* = 0.048), suggesting that higher maternal glucose levels may be associated with altered fetal hemodynamics. Also, the scatterplot of AoI PSV, S/D and MCA S/D according to HgA1c is shown in Fig. 4. It was examined whether there was a correlation between the AoI PI and maternal and perinatal characteristics. No statistically significant correlation was found in Table 4.

**Table 3:** Correlations between HbA1c with fetal doppler parameters

	***r***	***p***
*UA-S/D*	0.198	0.061
*UA-PI*	0.181	0.087
*MCA-PSV*	− 0.080	0.454
*MCA-S/D*	**0.289**	**0.006**
*MCA-PI*	0.13	0.224
*UtA-S/D*	− 0.125	0.24
*UtA-PI*	− 0.045	0.675
*AoI-PSV*	**0.238**	**0.024**
*AoI-S/D*	**0.2**	**0.048**
*AoI-PI*	0.116	0.277
*AoI-Ta max*	0.129	0.226
*AoI-EDV*	0.022	0.835
*AoI-RI*	0.175	0.098
*IFI*	− 0.175	0.098

*A p value of* < 0.05 indicates a significant difference and statistically significant p-values are in bold

r: Pearson’s correlation coefficient with 95% confidence interval, *HbA1c* hemoglobin A1c, *UA* umbilical artery, *MCA* middle cerebral artery, *UtA* uterine artery, *IVC* inferior vena cava, *AoI* aortic isthmus, *S/D* systolic/diastolic ratio *PI* pulsatility index, *RI* resistive index, *PSV* peak systolic velocity, *Ta max* time-averaged maximum velocity, *EDV* end diastolic velocity, *IFI* aortic isthmus flow index

**Table 4:** Spearman's correlation between aortic isthmus PI and maternal-perinatal characteristics

	***r***	***p***
*Maternal age*	0.034	0.662
*BMI*	0.04	0.608
*Parity*	0.142	0.069
*Gestational age at delivery*	− 0.034	0.666
*Birth weight*	0.066	0.401
*HbA1c*	0.116	0.277
*NICU admission*	− 0.089	0.257
*APGAR Score at 1st minute*	0.074	0.346
*APGAR Score at 5th minute*	0.109	0.162

A p value of < 0.05 indicates a significant difference

*PI* pulsatility index,* BMI* body mass index,* HbA1c*hemoglobin A1c,* NICU*: neonatal intensive care unit

**Fig. 5:**
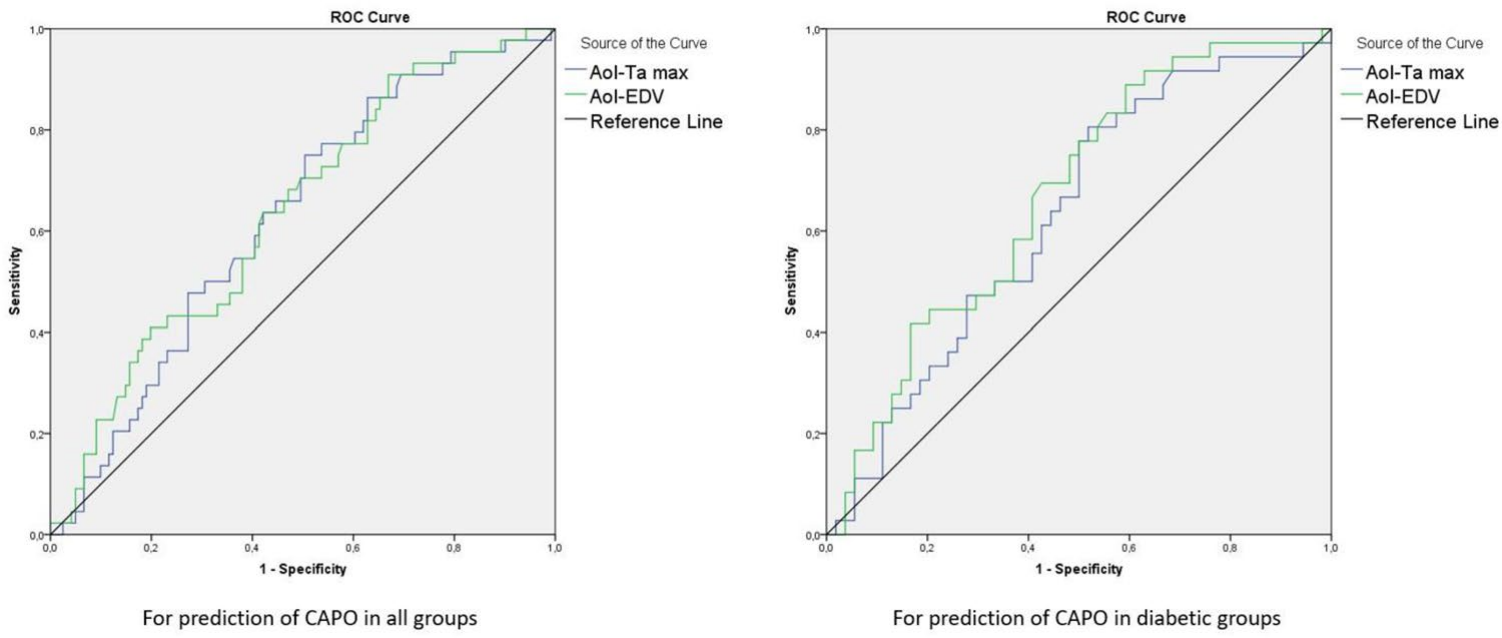
ROC curves of aortic isthmus time-averaged maximum velocity (AoI Ta-max) and End-Diastolic Velocity (AoI EDV) for the prediction of CAPO in all groups and in diabetic pregnancies

For the prediction of composite adverse perinatal outcomes, both the AoI Ta-max and EDV showed modest discriminative ability. In the entire cohort, AUC values were 0.627 for Ta-max (cut-off > 31.01, p = 0.013) and 0.638 for EDV (cut-off > 10.76, p = 0.007). Similar results were obtained in the diabetic subgroup (AUC = 0.630 and 0.669, respectively) (Table 5 and Fig. 5). In univariate analysis, gestational age at delivery was significantly associated with CAPO (OR 0.935, 95% CI 0.890–0.982; p = 0.008). This association remained significant in multivariate analysis after adjustment for maternal age, body mass index, and HbA1c (aOR 0.941, 95% CI 0.895–0.989; p = 0.018), whereas maternal age, body mass index, and HbA1c were not independently associated with CAPO (Table 6).

**Table 5:** Evaluation of Ta-max of aortic isthmus and End Diastolic Velocity of aorta in all groups and diabetic groups for prediction of composite perinatal adverse outcome by using ROC analysis

	LR +	LR-	Cut-off*	Sensitivity	Specificity	AUC	%95 CI	P-value
*AoI-Ta max (in all groups)*	1.51	0.63	> 31.01	63.6%	57.9%	0.627	0.54–0.72	**0.013**
*AoI-EDV (in all groups)*	1.51	0.63	> 10.76	63.6%	57.9%	0.638	0.55–0.73	**0.007**
*AoI-Ta max (in diabetic groups)*	1.43	0.68	> 31.59	61.1%	57.4%	0.630	0.51–0.75	**0.038**
*AoI-EDV (in diabetic groups)*	1.64	0.56	> 10.79	66.7%	59.3%	0.669	0.56–0.78	**0.007**

^*^Cut-off values were found according to Youden index

LR + :Positive likelihood ratio,* LR* Negative likelihood ratio,* AUC*Area under the curve,* CI*Confidence interval,* AoI*Aortic isthmus,* Ta-max*time-averaged maximum velocity,* EDV* end diastolic velocity

**Table 6:** Univariate and multivariate analysis of composite adverse perinatal outcome

	Univariate		Multivariate*	
	OR (95% CI)	*p value*	aOR (95% CI)	*p value*
*Maternal age*	0.939 (0.846–1.043)	0.244	0.959 (0.855–1.076)	0.478
*Body mass index*	1.007 (0.896–1.132)	0.911	1.004 (0.880–1.145)	0.953
*HgbA1c*	1.650 (0.988–2.758)	0.056	1.525 (0.866–2.685)	0.143
*Gestational age at delivery*	**0.935 (0.890–0.982)**	**0.008**	**0.941 (0.895–0.989)**	**0.018**

HbA1c: hemoglobin A1c, A p value of < 0.05 indicates a significant difference

^*^For multivariate analyses all variables were included in the model. Nagelkerke R Square: 0.236

## Discussion

This prospective case–control study investigated the clinical usefulness of AoI Doppler in fetuses with PGDM and GDM diagnosis, its correlation with other Doppler abnormalities and its relationship with perinatal outcomes.

Comprehending the relationship among maternal glycemic status, AoI Doppler parameters, and perinatal outcomes can provide critical insights for forecasting and addressing unfavorable outcomes in this high-risk pregnancies. The value of AoI Doppler has been thoroughly investigated in pregnancies complicated by fetal growth restriction (FGR), although its applicability in diabetes pregnancies is insufficiently examined [23–26].

Prolonged exposure to high blood glucose levels during pregnancy can lead to a decrease in the fetal heart structure, especially in the ability of the left ventricle to relax. This disrupts the flow of blood back to the heart, leading to pressure changes in the left atrium. As a result, the resistance encountered by blood passing through the pulmonary veins and foramen ovale may increase. In addition, the normal movement of the heart valves may be restricted, which reduces the amount of blood filling the left ventricle. To compensate for this, the fetal circulation increases right ventricular output; this compensatory response alters blood flow, particularly at the level of the AoI. These mechanisms have been demonstrated in studies [27, 28].

One of the most recent studies, conducted by Chen et al., demonstrated that GDM significantly alters fetal AoI Doppler parameters, providing valuable insights into the early effects of maternal diabetes on fetal circulation [24]. Chen et al. noticed no differences in umbilical blood flow parameters; however, the significantly lower aortic arch indices (IFI and ISI) in the GDM group indicate that hemodynamic alterations commence in the fetal aorta prior to influencing umbilical flow in pregnancies affected by GDM [24]. The study suggests that the combined evaluation of IFI and ISI may be a valuable tool in the early diagnosis of haemodynamic changes associated with GDM. In our study, which was encompassing both GDM and pregestational diabetic pregnancies, the IFI value, a parameter of the AoI, was lower than that of the control group; nevertheless, the difference lacked statistical significance. This may be ascribed to the comparatively limited sample size in our study.

In another study on this topic, Zielinsky et al. showed that the AoI IFI was significantly lower in the fetuses of PGDM and GDM mothers compared with the control group. It was emphasised that this change was due to impaired left ventricular diastolic function and that haemodynamic effects may occur early even in the absence of septal hypertrophy. This study demonstrates that independent of the increase in myocardial mass, maternal diabetes may cause other intrinsic changes in the fetal heart that affect ventricular distensibility and IFI may be a sensitive parameter in the early assessment of fetal cardiovascular effects of maternal diabetes mellitus [16]. The fact that the IFI value was lower in the foetuses of both pregestational and gestational diabetic pregnant women than in healthy foetuses was consistent with our study, but this lower IFI value was not significant in our study.

In a study by Lehtoranta et al. on fetal cardiovascular haemodynamics in pregnancies with T1DM, increased AoI pulsatility indices were found, supporting that AoI Doppler measurements may be an important parameter in the assessment of fetal cardiac load and circulatory changes due to diabetes [17]^(p1)^. In the present study, AoI PI values were higher in the PGDM group, but this increase was not statistically significant.

A recent systematic review and meta-analysis showed that retrograde AoI flow in pregnancies with FGR was strongly associated with adverse outcomes such as perinatal death, stillbirth and RDS. It has been emphasised that AoI flow may be an early indicator of fetal cardiac decompensation and hypoxia [26]. To our knowledge, no study has investigated the role of AoI Doppler parameters in predicting adverse perinatal outcomes in pregnant women with DM. While the literature often focuses on the assessment of fetal distress with CPR, MCA or UA Doppler parameters, the unique aspect of our study is the use of AoI in this context, which has been less studied. The AoI represents a critical transition point between the fetal cerebral and systemic circulation. In diabetic pregnancies, when glycaemic control is impaired, this balance is altered, resulting in disturbances in AoI flow patterns. In this study, significant correlations were observed between HbA1c levels and selected AoI Doppler parameters, indicating a potential association between maternal glycaemic status and fetal hemodynamic measures.

In addition, ROC analysis identified a threshold value of > 10.79 cm/s for AoI EDV. Although the sensitivity and specificity values were moderate, these findings suggest that AoI Doppler assessment may provide complementary information when integrated with established clinical and sonographic parameters for risk stratification and follow-up in high-risk diabetic pregnancies.

Although gestational age at delivery emerged as the dominant determinant of composite adverse perinatal outcome in multivariate analysis, this does not negate the potential clinical relevance of aortic isthmus Doppler assessment. Rather, AoI Doppler parameters may reflect subtle fetal hemodynamic adaptations that are not fully captured by conventional maternal or perinatal risk factors. While AoI indices did not function as independent predictors after adjustment, their associations with metabolic markers such as HbA1c and their modest ROC performance suggest that AoI Doppler provides complementary, pathophysiologically informative insight into fetal cardiovascular adaptation in diabetic pregnancies.

This study has several limitations. First, the relatively small sample size within each subgroup may limit statistical power and restrict the generalizability of the findings. Second, the single-center nature of the study may further limit external validity, and larger multicenter studies are warranted to confirm these results. Third, Doppler assessments were performed at a single time point, reflecting a cross-sectional evaluation of fetal hemodynamics rather than longitudinal changes across different trimesters.

The primary strength of this study is its prospective design, which enabled systematic and standardized data collection. In addition, the inclusion of both GDM and PGDM subgroups allowed a more comprehensive evaluation of perinatal outcomes across different diabetic phenotypes. The detailed and standardized Doppler methodology represents another important strength of the study. Furthermore, the assessment of clinically relevant perinatal outcomes enhances the translational value of the findings for maternal and fetal health. Finally, by focusing on aortic isthmus Doppler hemodynamics in diabetic pregnancies, this study addresses a genuine gap in the current literature.

## Conclusions

This study highlights the potential role of aortic isthmus Doppler assessment as an adjunctive tool in the evaluation of adverse perinatal outcomes in pregnancies complicated by diabetes mellitus. Further large-scale prospective studies are warranted to confirm these findings and to clarify their clinical relevance.

## Data Availability

The data that support the findings of this study are available from the corresponding author upon reasonable request.
